# Screening and Identifying a Novel ssDNA Aptamer against Alpha-fetoprotein Using CE-SELEX

**DOI:** 10.1038/srep15552

**Published:** 2015-10-26

**Authors:** Lili Dong, Qiwen Tan, Wei Ye, Dongli Liu, Haifeng Chen, Hongwei Hu, Duo Wen, Yang Liu, Ya Cao, Jingwu Kang, Jia Fan, Wei Guo, Weizhong Wu

**Affiliations:** 1 Liver Cancer Institute, Zhongshan Hospital, Fudan University, Key Laboratory of Carcinogenesis and Cancer Invasion, Ministry of Education, Shanghai 200032, China; 2 Department of Laboratory Medicine, Zhongshan Hospital, Fudan University, Shanghai 200032, China; 3 School of Life Sciences and Biotechnology, Shanghai Jiao Tong University, Shanghai 200240, China; 4 Shanghai Aijin Biochemical Science & Technology Co. Ltd., Shanghai 200336, China; 5 Cancer Research Institute, Xiangya School of Medicine, Central South University, Key Laboratory of Chinese Ministry of Education, Changsha 410078, China; 6 Shanghai Institute of Organic Chemistry, Chinese Academy of Sciences, Shanghai 200032, China; 7 Institute of Biomedical Sciences, Fudan University, Shanghai 200032, China

## Abstract

Alpha-fetoprotein (AFP) is a liver cancer associated protein and has long been utilized as a serum tumor biomarker of disease progression. AFP is usually detected in HCC patients by an antibody based system. Recently, however, aptamers generated from systematic evolution of ligands by exponential enrichment (SELEX) were reported to have an alternative potential in targeted imaging, diagnosis and therapy. In this study, AFP-bound ssDNA aptamers were screened and identified using capillary electrophoresis (CE) SELEX technology. After cloning, sequencing and motif analysis, we successfully confirmed an aptamer, named AP273, specifically targeting AFP. The aptamer could be used as a probe in AFP immunofluorescence imaging in HepG2, one AFP positive cancer cell line, but not in A549, an AFP negative cancer cell line. More interesting, the aptamer efficiently inhibited the migration and invasion of HCC cells after *in vivo* transfection. Motif analysis revealed that AP273 had several stable secondary motifs in its structure. Our results indicate that CE-SELEX technology is an efficient method to screen specific protein-bound ssDNA, and AP273 could be used as an agent in AFP-based staining, diagnosis and therapy, although more works are still needed.

Alpha-fetoprotein (AFP) is a major fetal plasma protein. Serum AFP is always low expressed in healthy adults, but often high expressed in nearly 75% hepatocellular carcinoma (HCC) patients with more than 500 ng/ml^1^. Since 1970 s, AFP has been used as the most important tumor biomarker for HCC diagnosis in clinically. Antibodies were usually used for AFP qualitative and quantitative assays with high sensitivity and specificity. However, some obvious defects, such as difficult producing and storage, high immunogenicity, easy degradation and low cell permeability have limited their use in a wide range. Therefore, a new reagent needs be developed as a surrogate in practice.

Aptamers are kinds of short single-stranded deoxyribonucleic acid (ssDNA) or ribonucleic acid (RNA) molecules, typically with 25–100 nucleotides^2^,^3^. They are able to bind a variety of targets such as proteins^4^, polypeptides^5^, metal ions^6^ and even living cells^7^ with high affinity, specificity and selectivity. Aptamers were screened by an *in vitro* selective method known as systematic evolution of ligands by exponential enrichment (SELEX) for the first time in 1990^2^,^3^. Briefly, a large initial library with up to 10^15^ different nucleic acids was used in the SELEX process and target-specific binding aptamers with high affinity and specificity were enriched during the repeated selection.

Similarly, aptamers can recognize target molecules using their different secondary or tertiary structures as antibodies do. The unique structures of aptamers contribute their high specificity against the target. More important, aptamers exhibit many superior advantages than antibodies: they can be largely, rapidly and automatically synthesized *in vitro*; they are stable and resistant to denaturation and degradation^8^; they are nontoxic and nonimmunogenic; they are easy to be chemically modified for heterogeneous diagnostic and therapeutic purposes^9^,^10^; and they show high affinities against target molecules with typical dissociation constants (K_d_) in the micromolar to picomolar range^11^. Therefore, aptamers are believed to have great application in cancer imaging, diagnosis and therapy.

In previous studies, several AFP-specific ssDNA and RNA aptamers with the K_d_ of 2.37 to 69.7 nM were successfully screened by SELEX/microfluidic chip or Ni-NTA agarose beads^12^,^13^. And the reported aptamers had a linear range from 12.5 to 800 ng/ml in AFP detection and exhibited inhibitory effects on HCC proliferation. However, SELEX/microfluidic chip needs expensive and unique equipment, while agarose beads selection is a time-consuming and arduous process. In this study, we present a SELEX strategy based on capillary electrophoresis (CE) to select AFP-specific ssDNA aptamers successfully. Furthermore, functional studies were confirmed that this kind of binding resulted in a robust suppression of HCC migration and invasion *in vivo*. All results suggest that AFP specific aptamers could be used potentially as a novel diagnostic and therapeutic agent in AFP positive HCC patients.

## Materials and Methods

### Chemicals, ssDNA and cell lines

The purified AFP protein (100 μg/ml) was purchased from Fitzgerald (Fitzgerald, MA, USA). Taq PCR MasterMix and 50 bp DNA Ladder were purchased from Tiangen (Tiangen Biotech Co., Ltd., Beijing, China). Dynabeads M-280 Streptavidin was purchased from Invitrogen (Invitrogen Life Technologies, Carlsbad, CA, USA). Bovine serum albumin was purchased from Sigma-Aldrich (Saint Louis, MO, USA). pMD^TM^ 19-T Vector Cloning Kit was purchased from Takara (TaKaRa Biomedicals, Shiga, Japan). Trans5α Chemically Competent Cell was purchased from TransGen (TransGen Biotech Co., Ltd., Beijing, China). All chemicals and reagents used were of analytical grade and prepared with deionized water.

The initial ssDNA library and the primers described in Table 1 were chemically synthesized by Sangon (Sangon Biotech Co., Ltd., Shanghai, China). The ssDNA library was dissolved in the CE Buffer (30 mM NaH_2_PO_4_) at a pH of 7.5.

Two human hepatoma cell lines, HepG2 and SMMC7721, and one human lung cancer cell line A549 were purchased from the Shanghai cell bank, Chinese Academy of Sciences. All these cells were cultured in Dulbecco’s modified eagle medium with high glucose (DMEM-H) (Gibco Life Technologies, Carlsbad, CA, USA) supplemented with 10% fetal bovine serum (FBS; Gibco).

### CE-SELEX Assays

AFP-bound ssDNA aptamers were selected and identified on the P/ACE MDQ CE system (Beckman Coulter, Inc., Fullerton, CA, USA) installed with 32 Karat software. CE was equipped with a PDA (Photodiode Array) detector set at 256 and 280 nm. Separations were performed on a polyvinyl alcohol-coated fused-silica capillary of 40 cm length, with an internal diameter of 50 μm. The detector window was set at 30 cm from the inlet. The capillary was pre-rinsed with deionized water at 20 psi for 5 min and then CE Buffer for the other 5 min. All experiments were carried out at 25 °C. Meanwhile, 10 μM of initial ssDNA library was incubated at 95 °C for 5 min and slowly cooled down at room temperature. Then the purified AFP protein was mixed with the ssDNA library at the final concentration of 50 μg/ml. The mixture were injected under the pressure of 0.5 psi during 10 s. Detection was in the anode for SELEX with a separation voltage set at −8 kV and a forward pressure assisted at 0.5 psi. AFP-bound aptamers were collected in a vial prefilled with 18 μl of deionized water (as the template of routine PCR). CE based selection of every round was separately performed three times to obtain sufficient active species.

### Generation of ssDNA sub-library

The AFP-bound ssDNAs were amplified by routine PCR. The reverse primer was labeled by biotin for ssDNA sub-library purification. The PCRs were set up in a final volume of 100 μl with 2 μl template described as above, 50 μl 2 × Taq PCR MasterMix, 0.4 μM P1 and 0.4 μM bio-P2. The reaction were performed at 94 °C for 3 min; then 10 cycles of 94 °C for 30 s, 58 °C for 30 s and 72 °C for 30 s; and finally at 72 °C for 5 min for extension.

Then ssDNA sub-library was separated from the PCR amplified products using a Dynabeads M-280 Streptavidin system according to the manufacturer’s instructions. About 10 μg of PCR products were incubated with 1 mg pre-washed Dynabeads for 20 min on a rotator. After three washing with B&W Buffer (5 mM Tris-HCl, 0.5 mM EDTA and 1 M NaCl at pH 7.5), the dsDNAs adsorbed on the Dynabeads were denatured with 100 μl of freshly prepared 150 mM NaOH for 2 min. The dynabead-unbound ssDNA was precipitated by absolute ethyl alcohol, resuspended in 20 μl of deionized water and electrophoresed on 8% polyacrylamide/7 M urea gels. PCR product and purified ssDNA were stained by GelRed (Biotium, Hayward, CA, USA) and quantified at 260 nm absorption. The remaining ssDNA pool was ready for use of next round selection.

### Cloning and sequencing of ssDNA

The 4th round ssDNA were amplified by PCR with unmodified primers (P1 and P2) at the same condition. The PCR products were purified with QIA quick PCR purification kit, cloned into the pMD 19-T vector and then transformed into Trans5α Chemically Competent Cell according to the manufacturer’s protocols. Ninety-seven white clones were picked out for nucleic acid sequencing by Sangon. Each was named as AP(XXX) based on their sequencing serial number.

### Validation of AFP-bound aptamers by CE

The primary homology and the secondary structure of all sequenced ssDNA were first predicted by DNAMAN software (version 5.2; Lynnon Co., Pointe-Claire, QC, Canada). And AFP-bound ssDNA candidates with a representative structure were selected and confirmed by CE with a laser-induced fluorescence (LIF) detector. Excitation was generated using the 488 nm line of an Ar + laser (Beckman Coulter) and emission was collected at 520 nm. Each candidate was labeled with 6-carboxy-fluorescein (6-FAM) at 5’ end. The mixtures with 10 μM FAM-labeled single aptamer and 100 μg/ml AFP were injected into the capillary and separated at the same CE condition. The bulk K_d_ of the aptamer to AFP was calculated using the equation 1, where [AFP]_tot_ is the total concentrations of AFP, [aptamer]_tot_ is the total concentration of aptamer, A1 and A2 are the areas of the peak of free aptamer and aptamer dissociated from the complex divided by the migration time of free aptamer and A3 is the area of the peak of the intact complex divided by the migration time of the complex^6^,^14^,^15^.


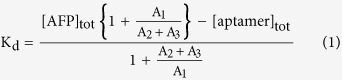


### Aptamer-based immunofluorescence imaging

AFP positive HepG2 cells and AFP negative A549 cells were seeded into 24-well plates (Corning, NY, USA) at an initial number of 2 × 10^4^ cells per well. After a 24-h culture, the cells were fixed with 4% paraformaldehyde for 30 min, then washed with PBS and blocked with 5% BSA for 60 min. Subsequently, the cells were incubated with 500 nM (10 μg/ml) FAM-labeled aptamer in 1% BSA for 60 min, washed with PBS again and then stained with 4’, 6-diamidino-2-phenylindole (DAPI; Invitrogen) for 90 s. Meanwhile, immunofluorescence staining using anti-AFP antibody (mouse anti-human; 10 μg/ml; Abcam, Cambridge, MA, USA) was performed as a positive control.

### Cell migration and invasion assays

Aptamers were transfected into HepG2, SMMC7721 and A549 cells at a final concentration of 100 nM in 24-well plates using Lipofectamine 2000 (Invitrogen) according to the product manual. In brief, cell migration and invasion were analyzed by a Transwell Permeable Supports system with 8-μm pores (Corning). For motility assays, 3 × 10^4^ cells were seeded into upper uncoated inserts; for invasion assays, 5 × 10^4^ cells were seeded into upper inserts with a Matrigel-coated membrane (BD, Franklin Lakes, NJ, USA). Cells were seeded in 1% serum medium and translocated to 10% serum media for 24 or 48 h. After discarding the non-migrating or non-invading cells, the remaining cells were fixed, stained, and analyzed by inverted microscopy.

### Motif, 3D Structure and binding mode predictions

Motifs in the sequence of aptamers were discovered using the server of Multiple Em for Motif Elicitation Ver 4.9.1 (MEME; http://meme.nbcr.net/meme/cgi-bin/meme.cgi)^16^. MEME usually finds the most statistically significant (low E-value) motifs first. The E-value of a motif is based on its log likelihood ratio, width, sites, the background letter frequencies and the size of the training set. Three-dimensional ssDNA structures were predicted and folded with iFoldRNA Ver 2.0 (http://troll.med.unc.edu/ifoldrna.v2/index.php)^17^,^18^, a novel web-based methodology for RNA structure prediction with near atomic resolution accuracy and analysis of RNA folding thermodynamics. Just like RNA, the ssDNAs were single-stranded. Therefore, before structural prediction, DNA sequences were first transformed to the corresponding RNA sequences, and later re-transformed again. Docking between aptamer and AFP protein was conducted using ZDOCK server Ver 3.0.2 (http://zdock.umassmed.edu/)^19^,^20^, which predicted the interacting models by the fast Fourier transform and utilized a combination of shape complementarity, electrostatics and statistical potential terms for scoring. Additionally, homology modeling for AFP Protein was performed with SWISS-MODEL^21^.

### Statistical analysis

Data were analyzed using GraphPad Prism 5 software. Quantitative variables were expressed as means ± SD and analyzed by one-way ANOVA and Student’s t-test. Results were considered statistically significant at P < 0.05.

## Results

### AFP-bound aptamers successfully selected by CE-SELEX

**Figure 1:**
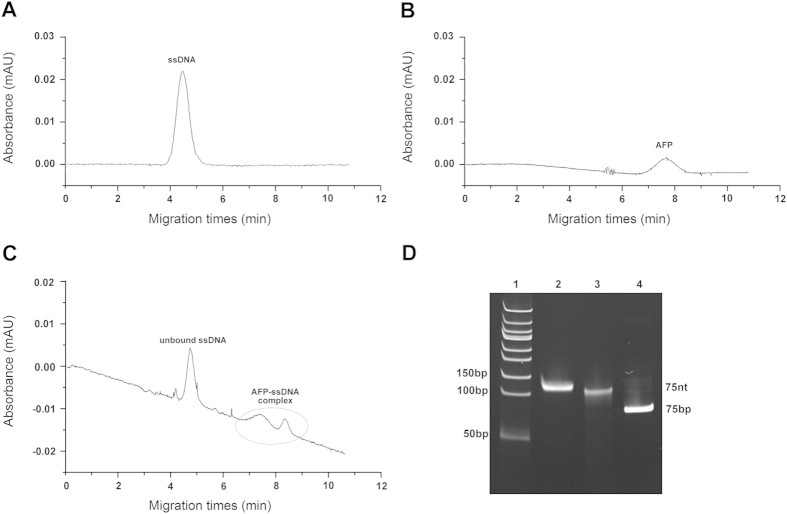
CE-SELEX selection and Electropherogram. (**A,B**) Capillary electropherogram of ssDNA library or AFP protein in free-solution, respectively. (**C**) Capillary electropherogram of separated unbound ssDNA and AFP-bound ssDNA. (**D**) Identification of ssDNA and PCR products using 8% PAGE/7 M urea gel electrophoresis. Lane 1: 50 bp DNA Ladder; lane 2: ssDNA sub-library generated by streptavidin-coated magnetic beads; lane 3: initial library; lane 4: PCR products. The shift of ssDNA should refer to the position of initial library, which is different from dsDNA.

A CE-SELEX process was recruited for isolating AFP-bound aptamers as described in the Material and Method. For reference, the denatured ssDNA or purified AFP protein was first electrophoresed by CE in free-solution and eluted at 4–5 min (Fig. 1A) or 7–8 min (Fig. 1B) respectively. Then, the denatured ssDNAs were mixed with AFP protein and separated by CE at the same condition. Two different fractions were detected in the eluent (Fig. 1C). One had a similar peak at 4–5 min and the other had a more complex peak at 7–8 min, indicating that the formation of complex with AFP altered the mobility of the ssDNA at a different electrophoresis rate. Therefore, certain ssDNAs had binding capabilities with AFP protein and the bound ssDNA can be easily separated by CE-SELEX method.

To enrich the targeted ssDNA aptamers, the fraction of AFP-bound peak was collected as a template and amplified by a routine PCR. After amplification, the band of double stranded DNA was stained at 75 bp. And then, the targeted ssDNA was successfully separated by Dynabeads through the interaction between biotin and streptavidin. The concentration and the yield of obtained ssDNA reached to approximately 2 μM and 20 μg. Furthermore, gel electrophoresis also showed that the ssDNA sub-library was purified and abundant enough for next round selection (Fig. 1D). During each round of selection, dozens of nanoliter products were collected into the vial. Over the 4 rounds of selection, the ratio of protein to ssDNA library was gradually lowered from 1:1 to 1:9.

### Specific AFP-bound aptamers confirmed by CE

**Figure 2:**
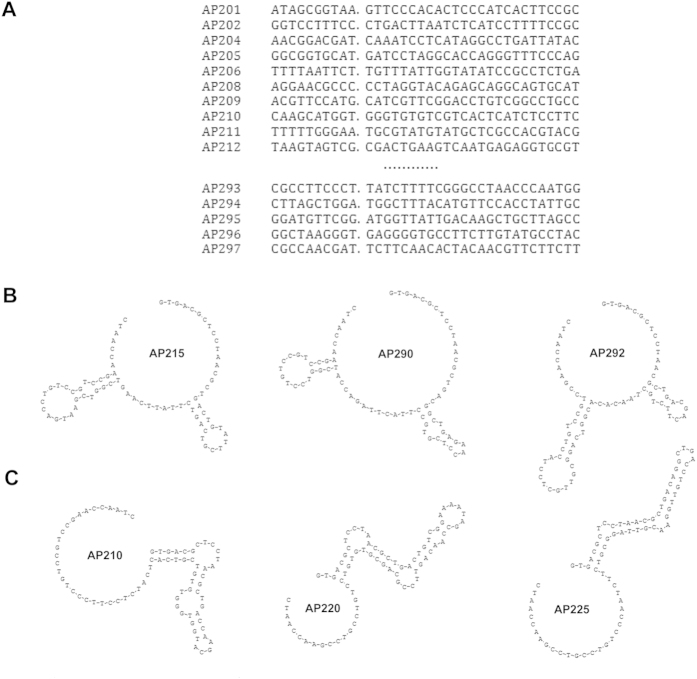
Primary and secondary structure of isolated aptamers. (**A**) Multiple sequence alignments of random region from 83 isolated aptamers. The complete results were listed in supplementary. (**B,C**) Similar secondary structures among different aptamers. For example, AP215, AP290 and AP292 have one big circular structure with two small arms, while AP210, AP220 and AP225 have one circular structure with a long arm.

**Figure 3:**
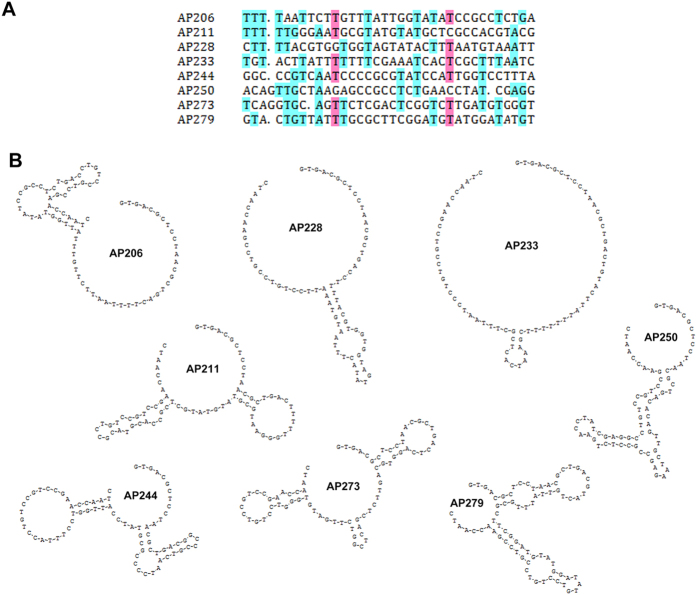
Eight characteristic secondary structures. (**A**) The multiple sequence alignments of random region from 8 representative secondary structures. More than 50% similarity with the consensus highlighted in light blue and more than 75% in magenta. (**B**) The distinct secondary structures of corresponding aptamers summarized from 83 sequencing aptamers.

**Figure 4:**
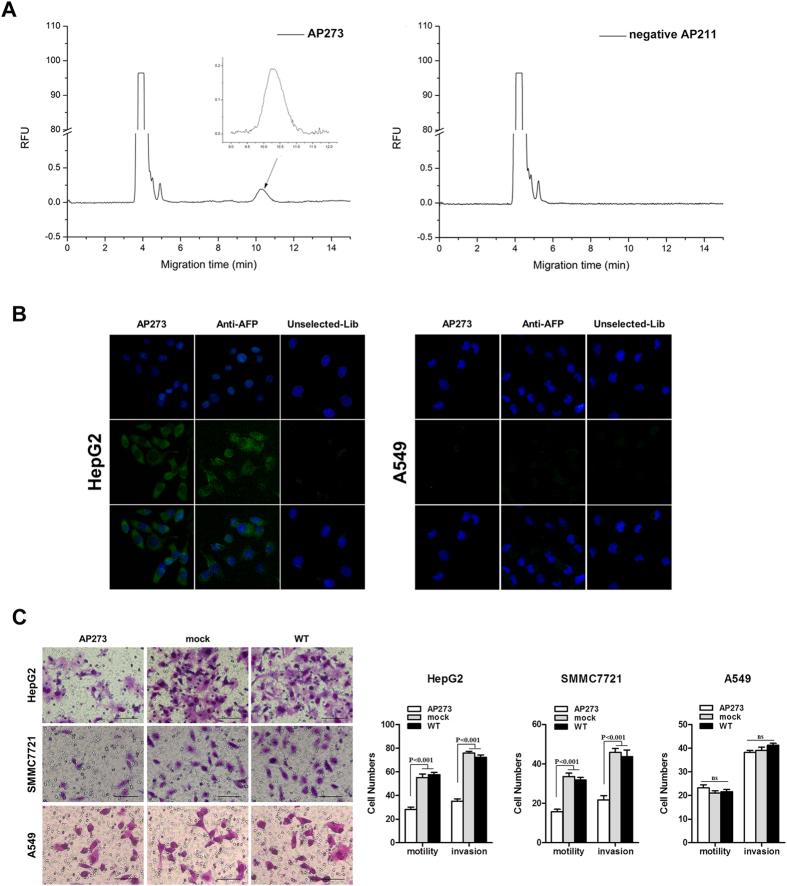
Binding specificity and functional evaluation of AP273. (**A**) Capillary electropherograms of FAM-labeled AP273 and AP211. AP273 specifically bound AFP, while AP211 did not in CE assays. (**B**) Fluorescence imagings of HepG2 and A549 cells stained by FAM-labeled AP273, anti-AFP antibody and FAM-labeled unselected aptamer library, respectively. About 10 μg/ml of aptamer or antibody as the final concentration was used. DAPI was used for contrast staining (blue). (**C**) Representative images (left) and statistical results (right) of cell migration and invasion. HepG2, SMMC7721 and A549 cells were transfected with AP273, AP211 (mock) or nontransfected (WT), respectively. Bars, 100 μm. Data are mean ± SD and representative of three independent experiments.

After 4 rounds of CE-SELEX selection, the AFP-bound fraction was amplified, purified, cloned and sequenced. Ninety seven clones were selected for DNA sequencing, and finally, 83 different nucleic acid sequences were available. By multiple sequence alignment and secondary structure analysis, these ssDNAs were varied from each other in primary sequences (Fig. 2A and Fig. S1), whereas some of them exhibited similar secondary structures. For example, AP215, AP290 and AP292 have one big circular structure with two small arms (Fig. 2B), while AP210, AP220 and AP225 have one circular structure with a long arm (Fig. 2C). For convenience, we picked out 8 different sequences with characteristic structures, including AP206, AP211, AP228, etc, for CE-based confirming assay. The primary and secondary structures of selected aptamers were summarized in Fig. 3. The AFP binding ability of each FAM-labeled aptamer was detected by CE based laser induced fluorescence detection system. Four of them, AP206, AP244, AP250 and AP273, were found with higher combining power to AFP, while the remaining had not, such as AP211 (Fig. 4A). These four positive aptamers showed similar binding affinity to AFP within a micromole range. Among them, AP273 had the highest affinity (K_d_ = 0.5 μM), which was much stronger than the second one AP244 (K_d_ = 4.8 μM).

### Immunofluorescence staining with FAM-labeled AP273

To confirm the targeted binding of AP273 against AFP, HepG2 and A549 cells were used and directly stained by FAM-labeled AP273. As expected, HepG2 was positively stained, while A549 is negatively. The staining results were confirmed once again by a specific AFP antibody based immunofluorescence assay (Fig. 4B). However, the image stained with FAM-labeled AP273 was more bright, homogeneous and accurate than these with AFP antibody and no specific staining was found in HepG2 using FAM-labeled initial ssDNA library. All these results implied that AP273 indeed specifically targets AFP protein *in vivo* and has superior permeability and intensity of fluorescence staining to AFP antibody.

### HCC migration and invasion suppressed by AP273

Naturally, we wonder next if there was any biological function of this specific binding. Two AFP expressed cells, HepG2 and SMMC7721, and one AFP negative cell A549 were recruited again. As there was almost no ssDNA transfecting protocol of living cells existed, we referred to the protocol of plasmid DNA transfection. Fortunately, both HepG2 and SMMC7721 cells were efficiently transfected with FAM-labeled AF273 according to their fluorescence intensity (data not shown). After transfected with AP273 at the final concentration of 100 nM, cell migration and invasion of both AFP expressed HCC cells were significantly suppressed compared with a mock aptamer AP211 (Fig. 4C). On the other hand, no obvious changes occurred in A549 cells. These results suggested that the specific AFP binding of AP273 did attenuate cell migration and invasion of AFP positively expressed cells.

### Predicting motif and 3D-structure of aptamer

**Figure 5:**
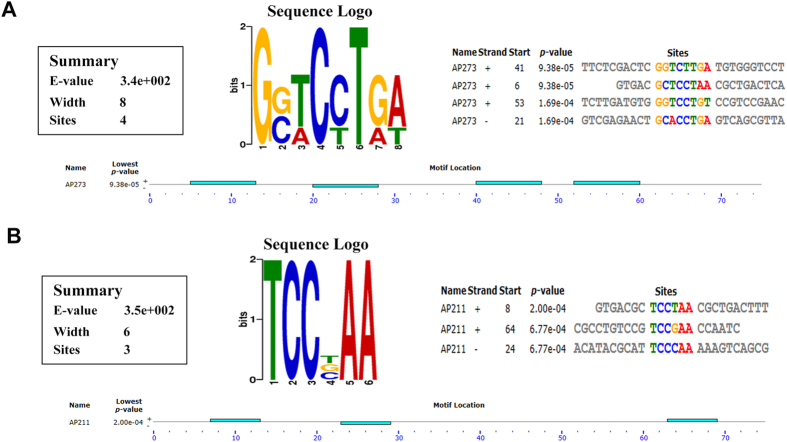
Motif prediction of AP273 and AP211. (**A,B**) Motifs of AP273 and AP211. Each light blue block represents a motif. The E-value is the statistical significance of the motif. ‘Sequence Logos’ contains stacks of letters at each position in the motif. The height of the individual letter in a stack represents the probability of defined nucleic acid at that position multiplied by the total information content of the stack. The sites are shown aligned with each other. Each site is identified by the name, the strand (if both strands of DNA sequences are being used, ‘-’ means the reverse complement of the sequence) and the position where the site begins. The p-value gives the probability of a random string (generated from the background letter frequencies) having the same match score or higher.

**Figure 6:**
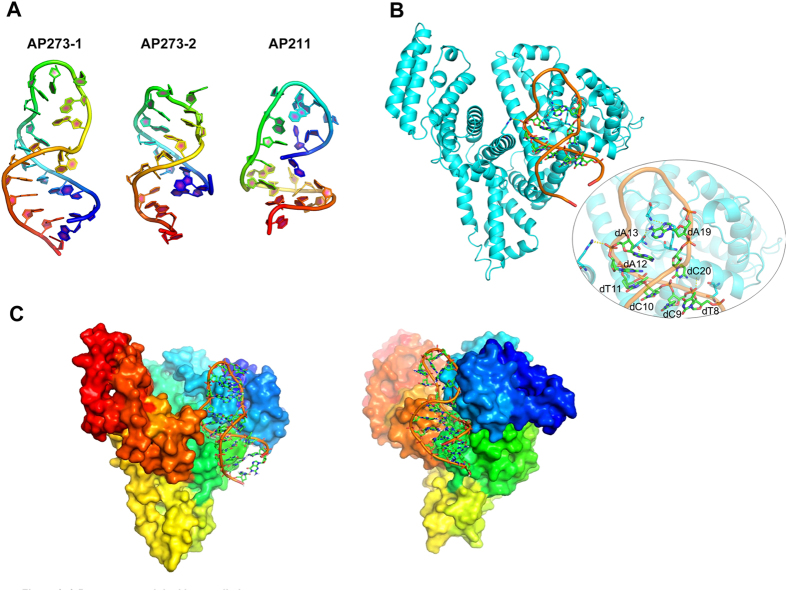
3-D structure and docking prediction. (**A**) Three-dimensional structures of AP273 and AP211. The motifs of AP273 can be folded into 2 kinds of structures, AP273-1 and AP273-2. (**B**,**C**) A docking model between AP273-1 and AFP protein. In particular, dC9, dC10, dT11, dA13, dA19, dC20 are in close contact with AFP. C, T and A represent the bases and the number represents the location of the base in the ssDNA sequence. (**C**) A 3-D interacting mode observed from two views.

To elucidate the effect of motif on target combining, AP273 and AP211, which were experimentally confirmed with positive and negative AFP-bound ability respectively, were used as the prototypes of motif analysis by MEME Tools. The results showed that several motif blocks were found in these two aptamer sequences (Fig. 5). AP273 contained longer interacting motifs, while AP211 only had scattered and shorter motifs. For AP273, 3 conserved sequences were found in motif ‘G[G/C][T/A]C[C/T]T[G/A][A/T]’ with the sequence of ‘GCTCCTAA’ starting at +6 position, ‘GGTCTTGA’ at +41 position and ‘GGTCCTGT’ at +53. Meanwhile, motif “TCC[T/G/C]AA” was found in the sequence of AP211 including the sequence of ‘TCCTAA’ at + 8 and ‘TCCGAA’ at +53. Furthermore, 3-D structures of these motifs were further analyzed by iFoldRNA Tools. The two tertiary structures of AP273 displayed much more helix and formed a tight structure than that of AP211 (Fig. 6A). The latter revealed an incomplete helix with loose structure. These data manifested that AP273 had a more characteristic and stable structure than the one of AP211, and even more, the well-helical structure may be important in the protein recognition. Hence, taking the first motif fragment in AP273 (AP273-1) as an example, we performed macromolecule docking between AP273-1 and AFP protein with ZDOCK server (Fig. 6B,C and Supplemental Movie). In this predicted binding mode, the helix of AP273-1 was properly embedded in the domain of AFP and dC9, dC10, dT11, dA13, dA19, dC20 were in close contact with protein, representing their key roles in the specific recognition with AFP.

## Discussion

HCC is the fifth common malignancy and the third common cause of cancer death globally^22^. In clinic, serum AFP is used as a main biomarker for HCC diagnosis^23^. In traditional detection, AFP is usually measured by antibody system, so that a lot of antibodies against AFP have been raised. Although most of them are sensitive and specific enough to meet their routine application, the arduous producing procedure, poor permeability and unstable characteristic reveal that antibody based detection system is not an optimal choice in *in-vivo* imaging, especially for intracellular proteins. Aptamers, composed of single-stranded DNAs or RNAs, are often folded into a tight structure in nature with low molecular weights, and recognize target molecule in a similar way of antibody by virtue of their complementary structures.

In this study, we applied CE-SELEX method to generate AFP novel probes. A 75-nt ssDNA with 35 randomized inserts will generate 4^35^ (about 10^22^) kinds of ssDNAs in theory, however, only give us 10^14^–10^15^ random ssDNAs in limit of synthetic technology^24^. After each CE-SELEX selection, the variety of input ssDNA library was gradually decreased and, meantime, the specific targets were enriched. As human IgE aptamer was succeeded in screen with only two-round running by Mendonsa and Bowser for the first time^25^,^26^, AFP-specific ssDNA aptamers were also successfully screened in a four-round selection in our system. The results once more indicated that CE-SELEX strategy is an efficient, high-resolving and time-saving one in aptamer screening.

After cloning and sequencing, 83 different sequences were finally obtained. Sequence alignment analysis revealed that these ssDNAs were heterogeneous and had no conserved nucleotide in their primary structures. It could be due to the high sensitivity of CE-SELEX that most AFP-targeted molecules with different affinity and binding sites were simultaneously screened and, subsequently, amplified by our PCR system. Although no homology in primary structure, some aptamers exhibited a similar secondary structure among them.

As advanced structures may exert determined effects on target binding, a total of 8 typical structures were summarized from 83 aptamers, and then randomly picked out one from every category for further identification. Using fluorescence detection system, 4 of 8 FAM-labeled aptamers were finally confirmed by laser-CE. Among them, AP273 has the highest affinity against AFP (K_d_ = 0.5 μM). Immunofluorescence staining also confirmed its specific binding with AFP in HepG2 cell. However, the K_d_ of AP273 was much lower than that of the reported ssDNA aptamer (AP-Taiwan, K_d_ = 2.37 nM)^12^. So, we in parallel compared the affinity of AP273 with AP-Taiwan using CE assay as well as a microplate testing system like ELISA^27^. Our preliminary results showed that the K_d_ values of aptamer AP273 and AP-Taiwan with AFP protein were 17.41 ± 4.32 nM and 18.89 ± 5.44 nM, respectively, by further calculations, and no significant difference was found between AP273 and AP-Taiwan (Fig. S2). Although we don’t know the exact reasons, we are trying to improve AFP affinity and specificity of AP273 through base modification or substitution.

It has been reported that AFP plays an important role in HCC tumorigenesis and progression^28^,^29^,^30^. Lee, *et al.* showed that a specific RNA aptamer was able to remarkably suppress the promoting effects of AFP on HCC growth and oncogene expression^13^. A similar inhibition on cell migration and invasion in both HCC cells was also achieved in our system after AP273 treatment. However, not every AFP-bound aptamer, such as AP248 and AP250, was found to have similar functions on cell migration and invasion in HCC (data not shown), indicating that different aptamers possibly bound in distinct domains and mediated the disparity functions of targets.

As structure domains play a pivotal role in the recognition process of antibody, we wonder if there is any similar structure existed in aptamers. The motifs of two aptamers, AP273 and AP211, were further analyzed by MEME and iFoldRNA. As expected, AP273, an AFP-specific binding aptamer, contained a serious of characteristic motifs with a condensed molecular conformation, while AP211, a negative one, exhibited no characteristic motifs with loose molecular conformation. We also simulated the macromolecule docking between AP273-1 and AFP. The predicted probable recognition sites may be critical to recognize protein. These preliminary data revealed that motif analysis may pave a new way for binding prediction of a given aptamer, and structure modification and mimic were probable directly and easily designed by motif analysis even before function studies.

In conclusion, a number of AFP-targeting aptamers were efficiently screened by CE-SELEX method. A novel aptamer, AP273, showed an excellent AFP-bound specificity and could modulate cell migration and invasion of HCC. Therefore, AP273, a superior ‘artificial antibody’, could be used as a potential agent in HCC diagnosis and, thereafter, molecular target therapy, although the underlying mechanisms need be further established.

## Additional Information

**How to cite this article**: Dong, L. *et al.* Screening and Identifying a Novel ssDNA Aptamer against Alpha-fetoprotein Using CE-SELEX. *Sci. Rep.* **5**, 15552; doi: 10.1038/srep15552 (2015).

## Supplementary Material

Supplem entary Information

Supplemental Movie

## Figures and Tables

**Table 1:** DNA Sequences Used in This Work.

Sequence ID	Sequence (5′ → 3′)
library	5′-GTGACGCTCCTAACGCTGAC-N35-CCTGTCCGTCCGAACCAATC-3′
forward primer (P1)	5′-GTGACGCTCCTAACGCTGAC-3′
reverse primer (P2)	5′-GATTGGTTCGGACGGACAGG-3′
biotin-P2	5′-Bio-GATTGGTTCGGACGGACAGG-3′
