# Supplementary material for: Screening and Identifying a Novel ssDNA Aptamer against Alpha-fetoprotein Using CE-SELEX
Source: Sci Rep. 2015 Oct 26;5:15552. doi: 10.1038/srep15552 (PMC4620443; doi:10.1038/srep15552)

Screening and Identifying a Novel ssDNA Aptamer against Alpha-fetoprotein Using CE-SELEX

Lili Dong[1*], Qiwen Tan[2*], Wei Ye[3], Dongli Liu[1], Haifeng Chen[3], Hongwei Hu[4], Duo Wen[1], Yang Liu[1], Ya Cao[5], Jingwu Kang[6], Jia Fan[1, 7], Wei Guo[2 §], Weizhong Wu[1 §]

[1] Liver Cancer Institute, Zhongshan Hospital, Fudan University, Key Laboratory of Carcinogenesis and Cancer Invasion, Ministry of Education, Shanghai 200032, China.

[2] Department of Laboratory Medicine, Zhongshan Hospital, Fudan University, Shanghai 200032, China.

[3] School of Life Sciences and Biotechnology, Shanghai Jiao Tong University, Shanghai 200240, China.

[4] Shanghai Aijin Biochemical Science & Technology Co. Ltd., Shanghai 200336, China.

[5] Cancer Research Institute, Xiangya School of Medicine, Central South University, Key Laboratory of Chinese Ministry of Education, Changsha 410078, China.

[6] Shanghai Institute of Organic Chemistry, Chinese Academy of Sciences, Shanghai 200032, China.

[7] Institute of Biomedical Sciences, Fudan University, Shanghai 200032, China

***Lili Dong and Qiwen Tan contributed equally to this work.**

§Correspondence should be sent to Weizhong Wu.

Tel & Fax: +86-21-54237181; E-mail: wu.weizhong@zs-hospital.sh.cn

Correspondence may also be addressed to Wei Guo.

Tel & Fax: +86-21-64041990-2376; E-mail: guo.wei@zs-hospital.sh.cn

```
AP201      GTGACGCTCCTAACGCTGACATAGCGGTAA.GTTCCCACACTCCCATCACTTCCGCCCTGTCCGTCCGAACCAATC    75
AP202      GTGACGCTCCTAACGCTGACGGTCCTTTCC.CTGACTTAATCTCATCCTTTTCCGCCCTGTCCGTCCGAACCAATC    75
AP204      GTGACGCTCCTAACGCTGACAACGGACGAT.CAAATCCTCATAGGCCTGATTATACCCTGTCCGTCCGAACCAATC    75
AP205      GTGACGCTCCTAACGCTGACGGCGGTGCAT.GATCCTAGGCACCAGGGTTTCCCAGCCTGTCCGTCCGAACCAATC    75
AP206      GTGACGCTCCTAACGCTGACTTTTAATTCT.TGTTTATTGGTATATCCGCCTCTGACCTGTCCGTCCGAACCAATC    75
AP208      GTGACGCTCCTAACGCTGACAGGAACGCCC.CCTAGGTACAGAGCAGGCAGTGCATCCTGTCCGTCCGAACCAATC    75
AP209      GTGACGCTCCTAACGCTGACACGTTCCATG.CATCGTTCGGACCTGTCGGCCTGCCCCTGTCCGTCCGAACCAATC    75
AP210      GTGACGCTCCTAACGCTGACCAAGCATGGT.GGGTGTGTCGTCACTCATCTCCTTCCCTGTCCGTCCGAACCAATC    75
AP211      GTGACGCTCCTAACGCTGACTTTTTGGGAA.TGCGTATGTATGCTCGCCACGTACGCCTGTCCGTCCGAACCAATC    75
AP212      GTGACGCTCCTAACGCTGACTAAGTAGTCG.CGACTGAAGTCAATGAGAGGTGCGTCCTGTCCGTCCGAACCAATC    75
AP214      GTGACGCTCCTAACGCTGACTCACCGGGCC.TACCGAAATCTAGGCTGCCCACATGCCTGTCCGTCCGAACCAATC    75
AP215      GTGACGCTCCTAACGCTGACTGTATTCGTC.AGTCTTATTCAAGTCGGTCGAATGACCTGTCCGTCCGAACCAATC    75
AP216      GTGACGCTCCTAACGCTGACGGGAATAACG.TGTAACGTAATAGCTGGGTTCACCTCCTGTCCGTCCGAACCAATC    75
AP217      GTGACGCTCCTAACGCTGACCGCGAGCCAC.TGACGGACACTCGGAATCCATTATTCCTGTCCGTCCGAACCAATC    75
AP218      GTGACGCTCCTAACGCTGACCACACGTAGG.GACAACATCACATGGGCGTTAGATACCTGTCCGTCCGAACCAATC    75
AP219      GTGACGCTCCTAACGCTGACCCGCCGTTCG.ATCCTTGATGCCCTGTTGGATGTTTCCTGTCCGTCCGAACCAATC    75
AP220      GTGACGCTCCTAACGCTGACTGTCGGAAAA.TAGCCAACAGTTCCGCAGCGTGTGTCCTGTCCGTCCGAACCAATC    75
AP221      GTGACGCTCCTAACGCTGACAACACCCTAA.TACCCACGGGTTGCCATACGGGGGTCCTGTCCGTCCGAACCAATC    75
AP222      GTGACGCTCCTAACGCTGACGGCACGAAGA.TGCTGTCAGGCCCTTTGCGTGTCGACCTGTCCGTCCGAACCAATC    75
AP223      GTGACGCTCCTAACGCTGACGGGCGCATAT.AATTCGCGATGTCTGTGCAATGTCCCCTGTCCGTCCGAACCAATC    75
AP224      GTGACGCTCCTAACGCTGACCGTCAGTATG.GTTCTTGGAACTATGTGACTGCTGGCCTGTCCGTCCGAACCAATC    75
AP225      GTGACGCTCCTAACGCTGACAGGCTGACCT.GTTGGAACGTTAGGCGTCTTCTAACCCTGTCCGTCCGAACCAATC    75
AP226      GTGACGCTCCTAACGCTGACAGGTTGGGGA.TATCGACGTTCCCATAAATACTCGCCCTGTCCGTCCGAACCAATC    75
AP227      GTGACGCTCCTAACGCTGACCTGCGGGACG.TGGATTGGCGGATATTGCTCGTCCGCCTGTCCGTCCGAACCAATC    75
AP228      GTGACGCTCCTAACGCTGACCTTTTACGTG.GTGGTAGTATACTTTAATGTAAATTCCTGTCCGTCCGAACCAATC    75
AP229      GTGACGCTCCTAACGCTGACAGGCAGCCTA.GCGGCTCAGGACTACTCTGATTGCCCCTGTCCGTCCGAACCAATC    75
AP230      GTGACGCTCCTAACGCTGACGACACACCTT.CCAAGCCACCAGAACTGGATGTACTCCTGTCCGTCCGAACCAATC    75
AP231      GTGACGCTCCTAACGCTGACAAATGGGCAA.AGCGTCTAAAGAATCTTGGTAGGGTCCTGTCCGTCCGAACCAATC    75
AP232      GTGACGCTCCTAACGCTGACGGACCATATA.CGCGAACTTGACGACGTCTCTCGTCCCTGTCCGTCCGAACCAATC    75
AP233      GTGACGCTCCTAACGCTGACTGTACTTATT.TTTTTCGAAATCACTCGCTTTAATCCCTGTCCGTCCGAACCAATC    75
AP234      GTGACGCTCCTAACGCTGACCGGGCGCCAA.GTCATCGTGGTAGCCAGGCACGTGACCTGTCCGTCCGAACCAATC    75
AP236      GTGACGCTCCTAACGCTGACGTAGAGCAGG.GGGCCAAGAACTAGTCATATCGCCCCCTGTCCGTCCGAACCAATC    75
AP237      GTGACGCTCCTAACGCTGACGGTGGTAAGA.AGCTAGCTTACGGGCTTTGTTTTATCCTGTCCGTCCGAACCAATC    75
AP238      GTGACGCTCCTAACGCTGACCTGCGCTCGA.CGTAGTCCGCTCGGGAGTACCAGGCCCTGTCCGTCCGAACCAATC    75
AP239      GTGACGCTCCTAACGCTGACAGGGAGCACA.TCTTAAAGGCTGCCACGCCCGATCGCCTGTCCGTCCGAACCAATC    75
AP240      GTGACGCTCCTAACGCTGACTGAAAAGGGG.GTACCTTTAGGACTAGCGTCAGTGCCCTGTCCGTCCGAACCAATC    75
AP241      GTGACGCTCCTAACGCTGACCGGGGTGAAT.CTCATGCTTACGGGGTTCGTACTAGCCTGTCCGTCCGAACCAATC    75
AP242      GTGACGCTCCTAACGCTGACATAGCGCACT.GTAGTAGGGACACCACCTCCGCCAGCCTGTCCGTCCGAACCAATC    75
AP243      GTGACGCTCCTAACGCTGACTCGGGGACAT.ACGCGGGCGGAAGTGCAACGTGTGTCCTGTCCGTCCGAACCAATC    75
AP244      GTGACGCTCCTAACGCTGACGGCCCGTCAA.TCCCCGCGTATCCATTGGTCCTTTACCTGTCCGTCCGAACCAATC    75
AP246      GTGACGCTCCTAACGCTGACCAGCACTGTC.GGATTAGCCACTGTTAGTTGGAGCCCCTGTCCGTCCGAACCAATC    75
AP248      GTGACGCTCCTAACGCTGACTGTCGTTTGT.TTGAGTGGATATTTCTTCTCTTCCGCCTGTCCGTCCGAACCAATC    75
AP249      GTGACGCTCCTAACGCTGACGTAAAAGCAC.CCTTAAGTCAGAGTACATGCCAGGGCCTGTCCGTCCGAACCAATC    75
AP250      GTGACGCTCCTAACGCTGACACAGTTGCTA.AGAGCCGCCTCTGAACCTATCGAGGCCTGTCCGTCCGAACCAATC    75
AP251      GTGACGCTCCTAACGCTGACTCAGAACGAC.CCGGTTCTGCCTACTGGCTGGTGGGCCTGTCCGTCCGAACCAATC    75
AP252      GTGACGCTCCTAACGCTGACCAGCGTACTT.TTGGTTATTCACGAGACATAAGCCGCCTGTCCGTCCGAACCAATC    75
AP254      GTGACGCTCCTAACGCTGACTTTGGAGAGG.CGTCGCCATGTTCCACTTAGCGTGGCCTGTCCGTCCGAACCAATC    75
AP255      GTGACGCTCCTAACGCTGACTTTGGAGAGG.CGTCGCCATGTTCCGCTTAGCGTGGCCTGTCCGTCCGAACCAATC    75
AP256      GTGACGCTCCTAACGCTGACCAAGCTGCAT.AATTCCGTCAAATTCGGAAGGTAGGCCTGTCCGTCCGAACCAATC    75
AP257      GTGACGCTCCTAACGCTGACGGTCTGCAGG.TACCGGCCAGCTTGTTCAGACGACTCCTGTCCGTCCGAACCAATC    75
AP258      GTGACGCTCCTAACGCTGACGTGGGCGTCG.AATTGTCGGTCTGATCTGGTCGCACCCTGTCCGTCCGAACCAATC    75
AP260      GTGACGCTCCTAACGCTGACGAGTCAAGTT.ATGTCGTACAGCGGAGCGGTATTCCCCTGTCCGTCCGAACCAATC    75
AP261      GTGACGCTCCTAACGCTGACGGCGGGGACC.TTCGATCATACTTCGTTCGAATCTCCCTGTCCGTCCGAACCAATC    75
AP262      GTGACGCTCCTAACGCTGACCAGCGGGATT.ATTCCCTCTTTGGGGCGGATCGACACCTGTCCGTCCGAACCAATC    75
AP263      GTGACGCTCCTAACGCTGACGAGTTGACTA.CAGATGGGCGTTAGGGTAGCTTTACCCTGTCCGTCCGAACCAATC    75
AP264      GTGACGCTCCTAACGCTGACCATAAGACAT.AGTGAGCCCGGATTGTAGCCTGGGTCCTGTCCGTCCGAACCAATC    75
AP265      GTGACGCTCCTAACGCTGACTAACTGTGGT.CTAAGCGTGTCTCTGGCTCAGTATCCCTGTCCGTCCGAACCAATC    75
AP266      GTGACGCTCCTAACGCTGACGGGCCGCTTG.GATTACATGCCAGGAGCTTTAATTACCTGTCCGTCCGAACCAATC    75
AP267      GTGACGCTCCTAACGCTGACAATACCTGCG.TTGCTTCTCATGTTGGTCTTTTCACCCTGTCCGTCCGAACCAATC    75
AP268      GTGACGCTCCTAACGCTGACCTCTGTAAGA.AAGAGCGTTCCTACAGCCATTTCAGCCTGTCCGTCCGAACCAATC    75
AP270      GTGACGCTCCTAACGCTGACGGGAGATGGTGCTACATATTCATCTTGC.CAGCATCCCTGTCCGTCCGAACCAATC    75
AP271      GTGACGCTCCTAACGCTGACTGAGCAGTCC.AAGCCACTACATGTTCGGTATCTTTCCTGTCCGTCCGAACCAATC    75
AP272      GTGACGCTCCTAACGCTGACGGTATTCTTA.ACGTCCGGCTGTTAGTCTTATTGTTCCTGTCCGTCCGAACCAATC    75
AP273      GTGACGCTCCTAACGCTGACTCAGGTGCAG.TTCTCGACTCGGTCTTGATGTGGGTCCTGTCCGTCCGAACCAATC    75
AP274      GTGACGCTCCTAACGCTGACCTGGTGACCT.GCGTTTATATCAGTCACGCCGTCGCCCTGTCCGTCCGAACCAATC    75
AP276      GTGACGCTCCTAACGCTGACGCAGGGGCCA.GTTTTTGTACGTCATCTGGCCGGGCCCTGTCCGTCCGAACCAATC    75
AP277      GTGACGCTCCTAACGCTGACAACTGGGCTG.CGTCTTCCCCGTTCTCACTGCAATCCCTGTCCGTCCGAACCAATC    75
AP279      GTGACGCTCCTAACGCTGACGTACTGTTAT.TTGCGCTTCGGATGTATGGATATGTCCTGTCCGTCCGAACCAATC    75
AP281      GTGACGCTCCTAACGCTGACGATGGTCAGGACGGTGACTGGAATCACGTTGT.TGGCCTGTCCGTCCGAACCAATC    75
AP282      GTGACGCTCCTAACGCTGACGGCCCTAGTG.ACCTGGGGGTTCAGTTCTCGCTCAGCCTGTCCGTCCGAACCAATC    75
AP283      GTGACGCTCCTAACGCTGACCACGGCATTG.CCCGGAGCACTAGGGGTAGTCCTGGCCTGTCCGTCCGAACCAATC    75
AP284      GTGACGCTCCTAACGCTGACTGTGCACAGC.CTCATGCTCGCGCAACTCTAGGCAGCCTGTCCGTCCGAACCAATC    75
AP285      GTGACGCTCCTAACGCTGACCAGACATTTA.CTGGGATCGATAAGAATCGTCTGACCCTGTCCGTCCGAACCAATC    75
AP287      GTGACGCTCCTAACGCTGACCTTGTAGTCG.GTAGGGATGCGAGTACAGACTCAGCCCTGTCCGTCCGAACCAATC    75
AP288      GTGACGCTCCTAACGCTGACTGGCGTAACG.TATGCTGTCTGTCAAGTGTGCTCGCCCTGTCCGTCCGAACCAATC    75
AP289      GTGACGCTCCTAACGCTGACATAGGGGGCG.CGGTTAGCGTACTTTGAGTTGGAGGCCTGTCCGTCCGAACCAATC    75
AP290      GTGACGCTCCTAACGCTGACGGCTGAGAAC.CTCGTGCCTTACTTAGACCATCGGTCCTGTCCGTCCGAACCAATC    75
AP292      GTGACGCTCCTAACGCTGACGACTTCTGCT.AACACACGGTCAGGCGTTGCTCCTACCTGTCCGTCCGAACCAATC    75
AP293      GTGACGCTCCTAACGCTGACCGCCTTCCCT.TATCTTTTCGGGCCTAACCCAATGGCCTGTCCGTCCGAACCAATC    75
AP294      GTGACGCTCCTAACGCTGACCTTAGCTGGA.TGGCTTTACATGTTCCACCTATTGCCCTGTCCGTCCGAACCAATC    75
AP295      GTGACGCTCCTAACGCTGACGGATGTTCGG.ATGGTTATTGACAAGCTGCTTAGCCCCTGTCCGTCCGAACCAATC    75
AP296      GTGACGCTCCTAACGCTGACGGCTAAGGGT.GAGGGGTGCCTTCTTGTATGCCTACCCTGTCCGTCCGAACCAATC    75
AP297      GTGACGCTCCTAACGCTGACCGCCAACGAT.TCTTCAACACTACAACGTTCTTCTTCCTGTCCGTCCGAACCAATC    75
Consensus  gtgacgctcctaacgctgac                                    cctgtccgtccgaaccaatc
```

Supplemental Figure 1. Complete sequence alignment of 83 aptamers using DNAMAN

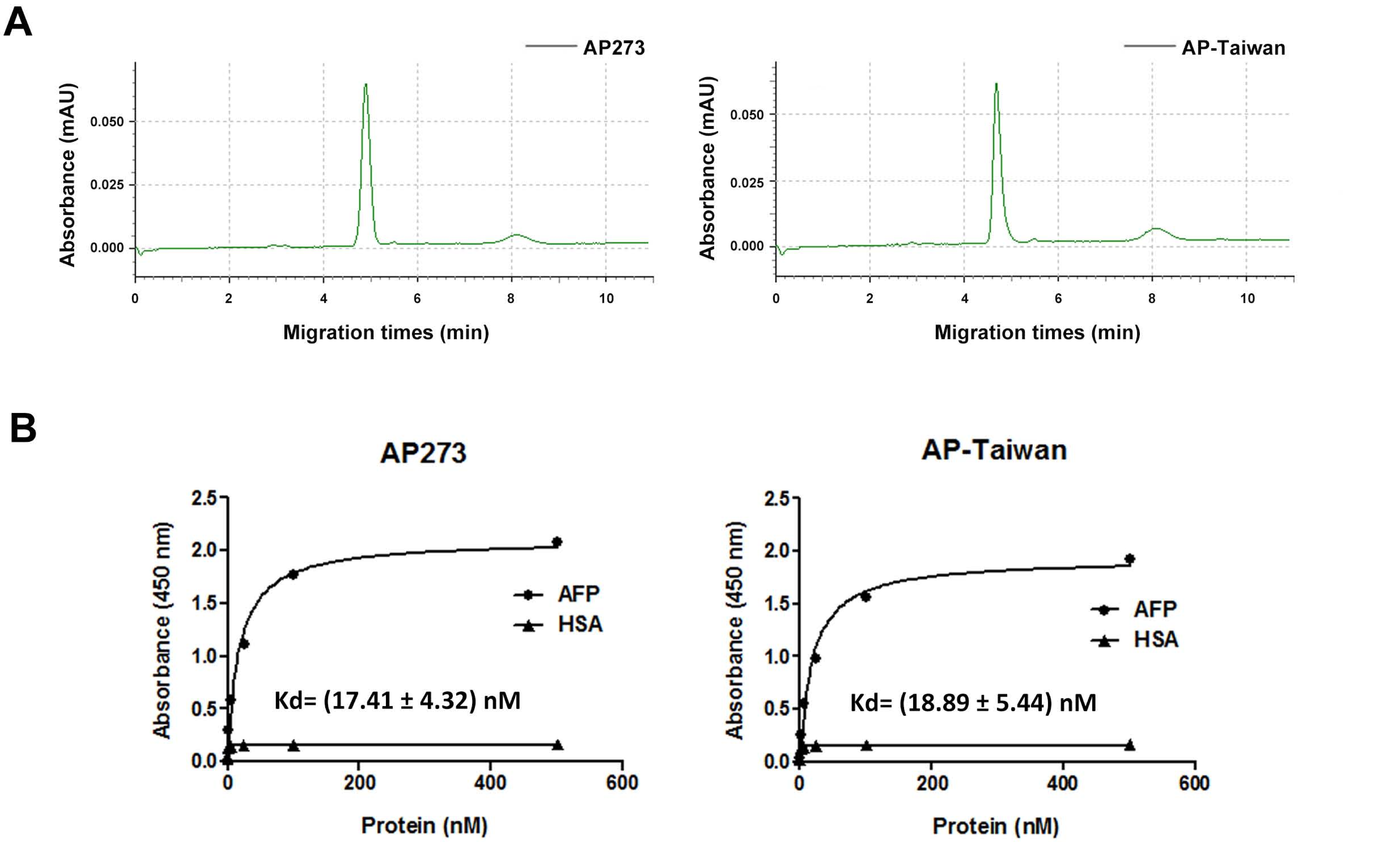

Supplemental Figure 2. Binding affinity evaluation of AP273 and AP-Taiwan

(A) Comparison between AP273 and AP-Taiwan using CE assays. Both of these aptamers run the similar peaks. (B) The microplate testing analysis of the comparison between AP273 and AP-Taiwan. The 96-well microplate was coated with AP273 or AP-Taiwan and pre-blocked with non-specific aptamers. A volume of 100 µl of AFP or human serum albumin (HSA) protein (as the negative control) of different concentrations (500 nM, 100 nM, 25 nM, 5 nM, 1 nM, 0 nM) was added to each well and incubated for 1h. After blocking with 3% BSA for 2 h, the plate was incubated with bio-labeled Anti-AFP antibody for 1 h. After washing 3 times, the streptavidin-horseradish peroxidase (SA-HRP) was added and incubated for 30 min, followed by washing. The plate was then added to substrate TMB for colorimetric detection on 450 nm. The titration curves were obtained based on above data and the equation Y = BmaxX / (Kd + X) was used to calculate the Kd according to GraphPad Prism 5.0. Y represented the mean value of OD450 nm, Bmax was the maximal value of OD450 nm, and X was the concentration of the protein. Compared to HSA, AP273 and AP-Taiwan all can bind AFP specifically and the Kd values with AFP were 17.41 ± 4.32 nM and 18.89 ± 5.44 nM, respectively.

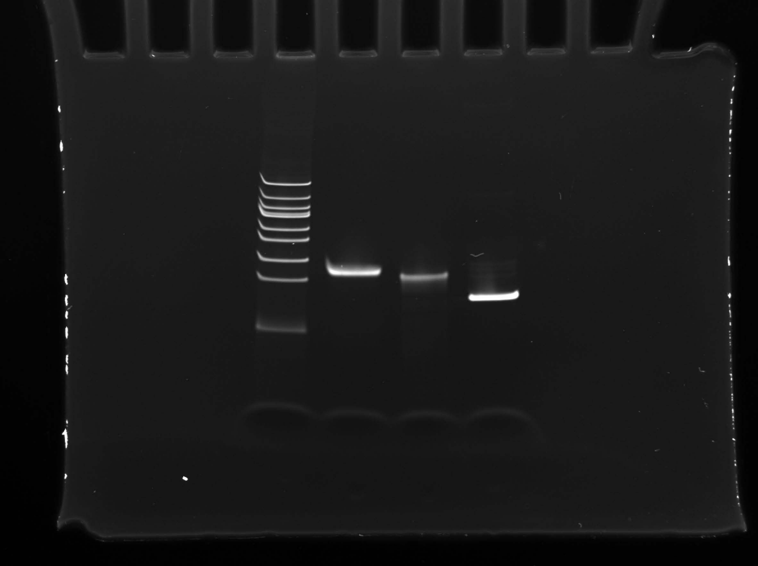

Supplement: Supplem entary Information [file srep15552-s1.pdf]
